# Mesenchymal stem cell transplantation ameliorates motor function deterioration of spinocerebellar ataxia by rescuing cerebellar Purkinje cells

**DOI:** 10.1186/1423-0127-18-54

**Published:** 2011-08-08

**Authors:** You-Kang Chang, Ming-Hsiang Chen, Yi-Hung Chiang, Yu-Fan Chen, Wei-Hsien Ma, Chian-You Tseng, Bin-Wen Soong, Jennifer H Ho, Oscar K Lee

**Affiliations:** 1 Institute of Clinical Medicine, National Yang-Ming University, Taipei, Taiwan; 2 Department of Radiation Oncology, Buddhist Tzu Chi General Hospital, Taipei Branch, New Taipei City, Taiwan; 3 School of Medicine, Tzu Chi University, Hualien, Taiwan; 4 Stem Cell Research Center, National Yang-Ming University, Taipei, Taiwan; 5 Department of Orthopaedic Surgery, National Yang-Ming University Hospital, Yi-Lan, Taiwan; 6 Department of Neurology, Taipei Veterans General Hospital, Taipei, Taiwan; 7 School of Medicine, National Yang-Ming University, Taipei, Taiwan; 8 Center for Stem Cell Research, Taipei Medical University-Wan Fang Medical Center, Taipei, Taiwan; 9 Graduate Institute of Clinical Medicine, Taipei Medical University, Taipei, Taiwan; 10 Department of Ophthalmology, Taipei Medical University-Wan Fang Medical Center, Taipei, Taiwan; 11 Department of Orthopaedics and Traumatology, Taipei Veterans General Hospital, Taipei, Taiwan

## Abstract

**Background:**

Spinocerebellar ataxia (SCA) refers to a disease entity in which polyglutamine aggregates are over-produced in Purkinje cells (PCs) of the cerebellum as well as other neurons in the central nervous system, and the formation of intracellular polyglutamine aggregates result in the loss of neurons as well as deterioration of motor functions. So far there is no effective neuroprotective treatment for this debilitating disease although numerous efforts have been made. Mesenchymal stem cells (MSCs) possess multi-lineage differentiation potentials as well as immuno-modulatory properties, and are theoretically good candidates for SCA treatment. The purpose of this study is to investigate whether transplantation of human MSCs (hMSCs) can rescue cerebellar PCs and ameliorate motor function deterioration in SCA in a pre-clinical animal model.

**Method:**

Transgenic mice bearing poly-glutamine mutation in ataxin-2 gene (C57BL/6J SCA2 transgenic mice) were serially transplanted with hMSCs intravenously or intracranially before and after the onset of motor function loss. Motor function of mice was evaluated by an accelerating protocol of rotarod test every 8 weeks. Immunohistochemical stain of whole brain sections was adopted to demonstrate the neuroprotective effect of hMSC transplantation on cerebellar PCs and engraftment of hMSCs into mice brain.

**Results:**

Intravenous transplantation of hMSCs effectively improved rotarod performance of SCA2 transgenic mice and delayed the onset of motor function deterioration; while intracranial transplantation failed to achieve such neuroprotective effect. Immunohistochemistry revealed that intravenous transplantation was more effective in the preservation of the survival of cerebellar PCs and engraftment of hMSCs than intracranial injection, which was compatible to rotarod performance of transplanted mice.

**Conclusion:**

Intravenous transplantation of hMSCs can indeed delay the onset as well as improve the motor function of SCA2 transgenic mice. The results of this preclinical study strongly support further exploration of the feasibility to transplant hMSCs for SCA patients.

## Background

Spinocerebellar ataxias (SCAs) are a group of inherited neurological disorders that are clinically and genetically very heterogeneous. They are progressive neurodegenerative diseases that are characterised by cerebellar ataxia, resulting in unsteady gait, clumsiness, and dysarthria. The cerebellar syndrome is often associated with other neurological signs such as pyramidal or extrapyramidal signs, ophthalmoplegia, and cognitive impairment [1]. Pathogenetic mechanism applies to SCAs caused by expansions of CAG repeats encoding polyglutamine tracts, as in the genes that underlie SCA1, SCA2, SCA3, SCA6, SCA7, SCA17, and dentatorubro-pallidoluysian atrophy, the so-called polyglutamine expansion SCAs [2,3]. Other SCA subtypes are caused by expansions in non-coding regions of genes for SCA8, SCA10, SCA12, and SCA31, and rare conventional mutations in SCA genes [2,3]. Mutant phenotype in the polyglutamine expansion SCAs has been widely considered to be primarily a result of a toxic gain-of-function in the mutant proteins in affected neurons [4,5]. Atrophy of the cerebellum and brainstem are most often the prominent features, but other structures can be affected, leading to a substantial range of phenotypes [5,6].

So far there is no cure of polyglutamine expansion SCAs although various therapeutic strategies have been postulated including silencing gene expression [7], increasing protein clearance, reducing the toxicity of the protein, influencing downstream pathways activated by the mutant protein and transplantation [4]. For symptom treatment, levodopa is temporarily useful for rigidity/bradykinesia and for tremor, and magnesium for muscle cramps in SCA2 patients [8], but neuroprotective therapy is not clinically available. In 1999, Low et al. reported that cerebellar allografts survived and transiently alleviated ataxia in a transgenic mouse model of SCA1 [9]. Subsequently, grafting murine neural precursor cells promoted cerebellar PCs survival and functional recovery in an SCA1 mouse model [10]. Murine MSCs (mMSCs) had been shown to be able to rescue PCs through releasing of neurotrophic factors and improve motor functions in a mouse model of cerebellar ataxia [11]. Although the surface phenotype and multilineage potential of mMSCs used in this study [11] was not demonstrated completely, these results suggested that MSC transplantation may be beneficial to SCA2 transgenic mice.

MSCs are defined as plate-adhering, fibroblast-like cells possessing self-renewal ability with the capacity to differentiate into multiple mesenchymal cell lineages such as osteoblasts, chondrocytes, and adipocytes. MSCs are easily accessible and isolated from a variety of tissues such as bone marrow, umbilical cord blood, trabecular bone, synovial membrane, and adipose tissue [12-16]. MSCs also provide the advantage of minimizing immune reactions because cells can be derived from the respective patient. Furthermore, several human trials of MSCs have shown no adverse reactions to allogenic MSC transplants [17,18]. Many studies show that systemically administrative hMSCs home to site of ischemia or tissue injury to repair injured tissues [19]. MSCs transplantation had been adopted in several clinical trials of neurological disease, including of multiple system atrophy [20], Parkinson's disease [21], amyotrophic lateral sclerosis [22], and ischemic stroke [23] with encouraging early or long-term results.

In our previous studies, we showed that clonally derived human MSCs (hMSCs), under chemically defined conditions, differentiate into neuroglial-like cells that not only express neuroglial-specific genes but also possessed a resting membrane potential and voltage-sensitive calcium channels on the membrane [13]. We also showed that in utero transplantation of hMSCs in mice contributed to numerous tissues, including the brain and spinal cord [24]. Donor hMSCs engrafted into murine tissues originating from all three germ layers and persisted for up to 4 months or more after delivery.

Therefore, the purpose of this study is to investigate whether transplantation of human MSCs (hMSCs) can rescue cerebellar PCs and ameliorate the deterioration of motor function in SCA in a pre-clinical animal model. Transgenic mice bearing poly-glutamine mutation in ataxin-2 gene (C57BL/6J SCA2 transgenic mice) were serially transplanted with hMSCs intravenously or intracranially before and after the onset of motor function loss. Motor function of mice was evaluated by an accelerating protocol of rotarod test every 8 weeks. Immunohistochemical stain of whole brain sections was adopted to demonstrate the neuroprotective effect of hMSC transplantation on cerebellar PCs and engraftment of hMSCs into mice brain.

## Materials and methods

### Culture of hMSCs

The isolation and characterization of hMSCs from bone marrow was performed as reported previously [25,26]. An approval from the Institutional Review Board of the Taipei Veterans General Hospital has been obtained prior to commencement of the study. hMSCs used in this study were clonally-derived, and their surface immune phenotype as well as multilineage differentiation potentials into osteoblasts, adipocytes, and chondrocytes were confirmed [25,26]. hMSCs of passage 8-10 were used for transplantation. Before transplantation, hMSCs were trypsinized with trypsin/EDTA 0.25%, counted for cell number and suspended in 80 μL PBS.

### Animal Model

C57BL/6J SCA2 transgenic mice were purchased from University of Texas Southwestern Medical Center (Dallas, Texas, USA) and wild-type C57BL/6J mice were purchased from Tzu Chi University Laboratory Animal Center (Hualien, Taiwan). All animal experiments were performed with the approval of the Animal Care Committee of the Taipei Veterans General Hospital.

### MSC Labeling with Superparamagnetic Iron Oxide (SPIO) nanoparticles for in vivo Cell Tracking

Amine (NH_3_^+^) surface modified iron-oxide nanoparticles of 6 nm diameter without polymer coating were prepared as reported previously [27]. hMSCs were seeded in culture plates at the density of 4 × 10^4 ^cells/well and were allowed for attachment and growth for 24 h. Before treatment, 50 μg/ml of SPIO were coated by mixing with 0.75 μg/ml poly-L-lysine (Sigma-Aldrich) in the culture medium at room temperature for 1 h. After that, hMSCs were incubated in SPIO-containing medium for 24 h. After labeling, the cultures were washed with PBS thoroughly to remove excess SPIO in the medium for further transplantation.

### MR Image of Mice after Intracranial SPIO-labeled hMSC Transplantation

Before intracranial transplantation, 100 μL trypan blue (Sigma-Aldrich) was injected through foramen magnum into position of cerebellum in a wild-type mouse, which was immediately sacrificed for visual examination of cerebellum to determine target accuracy. MR imaging was used to demonstrate the transplant site in living mice which received intracranial hMSCs transplantation. MR images of three mice were measured in a Bruker BioSpec 7T system (Bruker BioSpin MRI, Ettlingen, Germany). Mice were anesthetized, followed by injection of 8.4 × 10^6 ^per kg of mice body weight SPIO-labeled or unlabeled hMSCs in PBS through foramen magnum into cerebellum. Images were taken 24 h later under anesthesia using T2 weighted MR acquisition sequence with the following parameters: fast spin echo with TR/TE = 2500 ms/33 ms, ET = 10 ms.

### Intravenous and Intracranial hMSCs Transplantation

To evaluate the neuroprotective effects of hMSCs, 4.2 × 10^7 ^or 8.4 × 10^6 ^hMSCs per kg of mice body weight were injected via tail vein (IV hMSC-Tg group) or through foramen magnum into position of cerebellum (IC hMSC-Tg group) of C57BL/6J SCA2 transgenic mice. In IV hMSC-Tg group, hMSCs were transplanted at 12, 23, 33 and 42-week-old (n = 14). In IC hMSC-Tg group, hMSCs were transplanted at 12, 23, and 33-week-old (n = 5). Treated mice were compared to control SCA2 transgenic (Control-Tg) (n = 10) and wild-type (Control-Wt) (n = 16) mice.

### Motor Behavior Assessment: Accelerating Rotarod Test

Since 9 weeks of age, sex and weight-matched IV hMSC-Tg, IC hMSC-Tg, Control-Tg, and Control-Wt mice were tested on the rotarod (Singa Technology Corporation, Taipei, Taiwan) every 8 weeks, which underwent linear acceleration from 4 to 40 rpm in 300 seconds. Latency to fall from rotarod was recorded in seconds. Each trial lasted for a maximum of 5 min and mice were rested for minimum 15 min between trials to avoid fatigue. After rotarod test, the body weights of mice were recorded. Mice underwent three trials per day for four consecutive days, and the mean latency to fall of each day was considered for statistical analysis.

### Histological Examination and Immunohistochemistry: Purkinje Cells

Three mice from each group at > 50 weeks of age were sacrificed and processed for histological examination and immunohistochemistry (IHC) of the cerebellar PCs. Mice whole brain tissues were fixed in 3.7% formalin overnight after sacrifice under anesthesia and embedded selected samples in paraffin. Sections (4 μm) were cut and mounted onto microscopic slides. Sections were rehydrated by rinsing twice at 5 min intervals in xylene, 100% ethanol, 95% ethanol and 80% ethanol. After deparaffinization, sections were treated with 3% H_2_O_2 _for peroxidase inactivation, heated in 10 mM citrate buffer (with 0.05% Tween20) for antigen retrieval, blocked with 1% blocking solution (1% BSA and 0.1% Triton X-100 in PBS). Sections were incubated with anti-calbindin D-28K monoclonal antibodies (Sigma-Aldrich) diluted in blocking solution (1:300) for 40 min at room temperature (RT). After three extensive washes with PBS, sections were incubated with secondary antibody diluted in blocking solution (1:1000) for 40 min at RT. Primary antibodies were detected using DAB (3, 3'-Diaminobenzidine tetrahydrochloride) Two-component Enhanced Liquid Substrate System (Sigma-Aldrich), enhanced by DAB enhancer, and visualized with diaminobenzidine (DAB; Sigma-Aldrich). We counterstained with aqueous haematoxylin (Sigma-Aldrich). For direct comparison we processed all slides in a single batch to minimize variability.

### Count of Cerebellar Purkinje Cells

To determine whether MSC transplantation rescued PC loss in cerebellum of C57BL/6J SCA2 transgenic mice, we counted calbindin-D28K-positive PCs from twelve mice in IV hMSC-Tg, IC hMSC-Tg, Control-Tg, and Control-Wt group (three mice in each group). Every 8^th ^sections in the consecutive series of each mouse were selected and selected parasagittal sections were prepared for the counting from each mouse. Numbers of PCs under 20 100 × fields which randomly selected from non-concave area of parasagittal sections were counted and summed. Then average PC number of each mouse was calculated.

### Immunohistochemistry: hMSCs

Specific antibody which reacted with human beta2 microglobulin (Abcam, code: ab15976) was chosen to demonstrate hMSCs in murine brain tissue by IHC. The specificity of the antibody had been ascertained by crossed immunoelectrophoresis. Murine whole brain sections which processed for PCs counting were used for staining. Sections (4 μm) were cut and mounted onto microscopic slides. Sections were rehydrated by rinsing twice at 5 min intervals in xylene, 100% ethanol, 95% ethanol and 80% ethanol. After deparaffinization, sections were treated with 3% H_2_O_2 _for peroxidase inactivation, heated in 10 mM citrate buffer (with 0.05% Tween20) for antigen retrieval, and blocked with 1% blocking solution (1% BSA and 0.1% Triton X-100 in PBS). Sections were incubated with specific anti-human β2 microglobulin polyclonal antibodies (Abcam) diluted in blocking solution (1:400) for 40 min at RT. After three extensive washes with PBS, sections were incubated with secondary antibody diluted in blocking solution (1:1000) for 40 min at RT. Primary antibodies were detected using EnVision Detection System (DAKO), and visualized with diaminobenzidine (DAB; DAKO). We counterstained with aqueous haematoxylin (Sigma-Aldrich). For direct comparison we processed all slides in a single batch to minimize variability.

### Statistical analysis

Data are presented as the mean ± standard error of mean (SE) for at least three times of independent experiments. The results were compared using one-way ANOVA, Tukey's test as Post hoc test, and Student's T test. Statistical significance was determined at 95% confidence interval.

## Results

### Confirmation of Successful Intracranial Delivery of hMSCs

**Figure 1:**
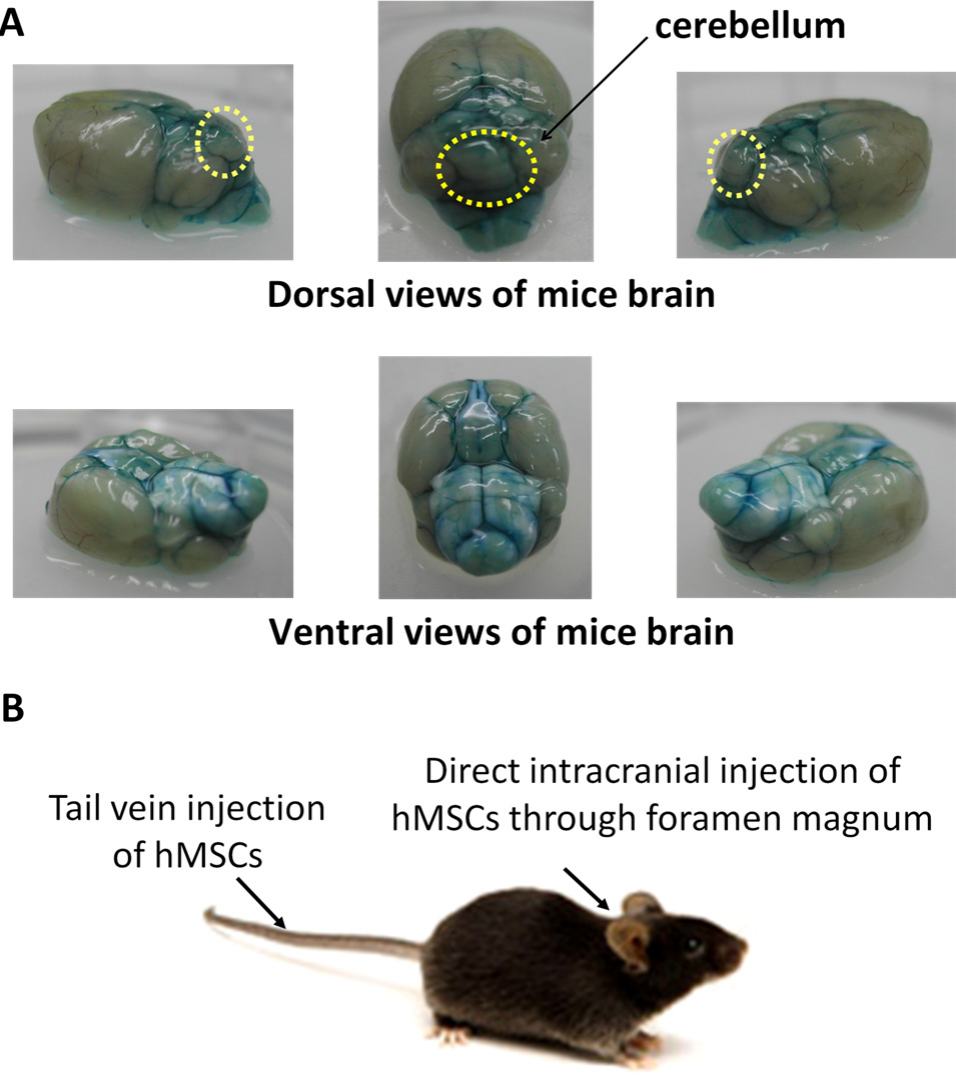
**Route of human mesenchymal stem cells transplantation and gross pictures of mice brain after trypan blue injection**. (A) 100 μL trypan blue was injected through foramen magnum into position of cerebellum in a wild-type mouse, which was immediately sacrificed for visual examination to determine target accuracy. Most of the areas staining by trypan blue were located at cerebellum, medulla and nearby regions. (B) hMSCs were injected intravenously via tail vein or intracranially through foramen magnum under anesthesia. hMSCs, human mesenchymal stem cells.

**Figure 2:**
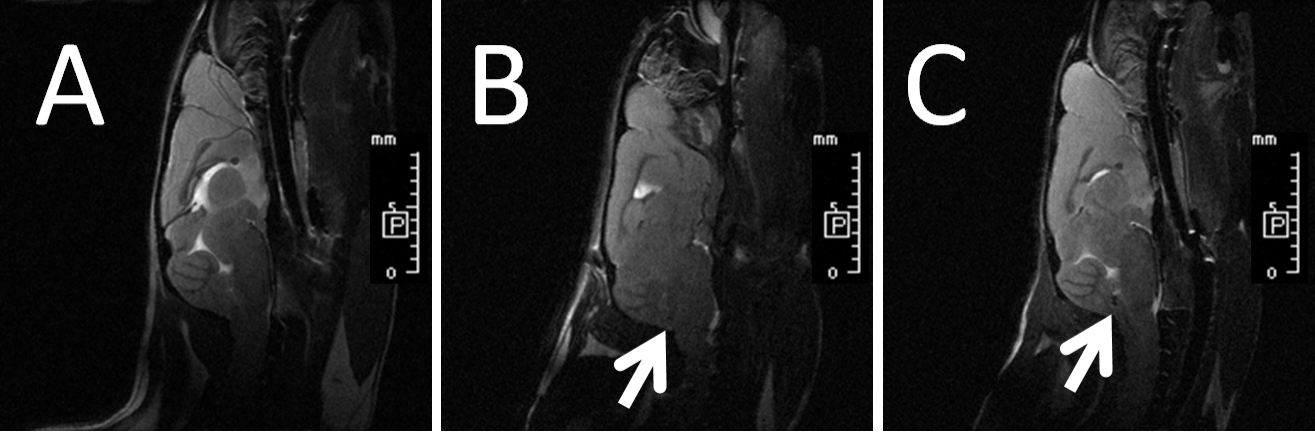
**Magnetic resonance images of mice after superparamagnectic iron oxide nanoparticles (SPIO)-labeled and unlabeled human mesenchymal stem cells transplantation**. Mice were anesthetized, followed by injection of 8.4 × 10^6 ^per kg of mice body weight unlabeled hMSCs (A, wild-type mouse) or SPIO-labeled hMSCs (B, wide-type mouse; C, SCA2 transgenic mouse) in PBS through foramen magnum intracranially, and then measured in a 7-T MR imager 24 h later. (A) No signal was detected in the medulla or cerebellum of wild-type mouse after intracranial transplantation of unlabeled hMSCs. (B) A significant decreased T2 signal intensity of the SPIO (white arrow) was detected in the dorsal site of medulla of wild-type mouse after intracranial transplantation of SPIO-labeled hMSCs. (C) A significant decreased T2 signal intensity of the SPIO (white arrow) was detected in the dorsal site of medulla of transgenic mouse after intracranial transplantation of SPIO-labeled hMSCs. The length of each small scale was 1 mm. The letter "P" indicated posterior direction.

Whole brain tissue of control mouse which was injected with trypan blue through foramen magnum into position of cerebellum was inspected after sacrifice, and most of the areas staining by trypan blue were located at cerebellum, medulla and nearby regions (Figure 1A). MR imaging was used to demonstrate the transplant site in living mice which received intracranial hMSCs transplantation. No decreased MRI signal intensity was observed in the medulla or cerebellums of wild-type mouse after intracranial injection of unlabeled hMSCs (Figure 2A). As shown in Figure 2B and 2C, a significant decreased T2 signal intensity was detected in the dorsal site of medulla, which was adjacent to cerebellums of wild-type and transgenic mice after intracranial injection of SPIO-labeled hMSCs. No evidence of major trauma or intracerebellar hemorrhage was detected in the medulla or cerebellums, either. These MR images further confirmed the injected hMSCs were located in the dorsal site of medulla, which was adjacent to cerebellum, and this invasive procedure didn't cause major trauma or intracranial hemorrhage at the injection site, as well as did not hamper the evaluation of motor function by rotarod test.

### Motor Behavior of SCA2 Transgenic Mice Improved after hMSC Transplantation Intracranial hMSC injection

**Figure 3:**
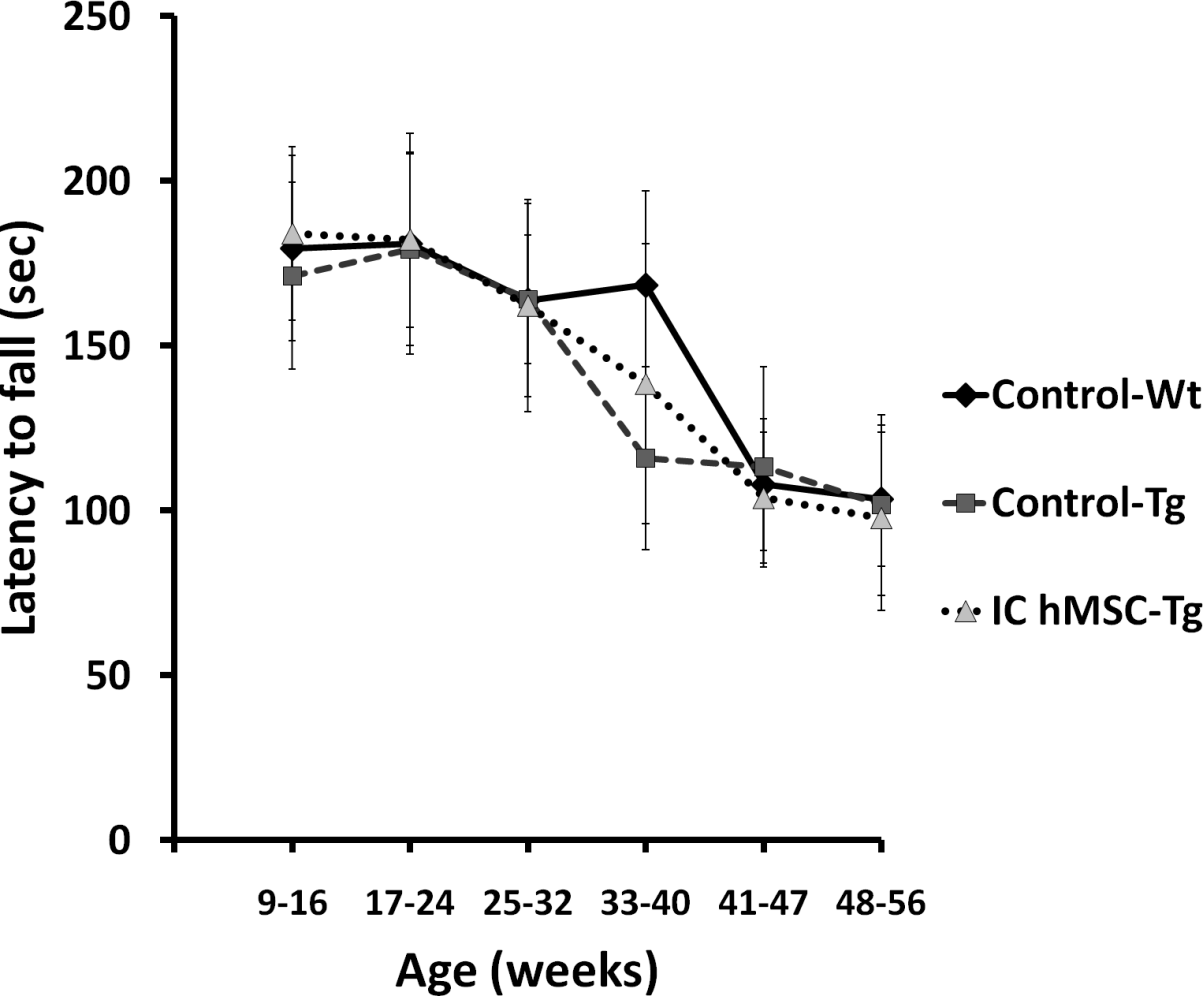
**Average of rotarod performance of mouse which received intracranial human mesenchymal stem cells transplantation at sequential periods**. Average of latency to fall from rotarod (in seconds) of mice after serial hMSCs implantation through intracranial injection was compared every 8 weeks. Rotarod performance of SCA2 transgenic mice (n = 5) was not significantly improved by serial intracranial hMSCs transplantation at 33-40 weeks of age (p = 0.055). hMSCs, Statistical analysis between each group was performed by one-way ANOVA (p = 0.055), and between Control-Wt (n = 16) and Control-Tg group (n = 10) was performed by t test (p < 0.05).

Rotarod testing showed that motor performance of SCA2 transgenic mice was not significantly different from that of wild-type mice at six weeks and transgenic mice started to perform poorly since 16 weeks of age with progressive deterioration from 26 weeks of age [28]. In our study, Control-Tg mice started to perform poorly since 25 weeks of age with progressive deterioration from 33 weeks of age (Figure 3) (t test, p < 0.05). SCA2 transgenic mice which received serial intracranial hMSC injection for three times had a trend of better rotarod performance than Control-Tg mice at 33-40 weeks of age, but the difference was not significant due to large error bar (one-way ANOVA, p = 0.055) (Figure 3).

### Intravenous hMSC injection

**Figure 4:**
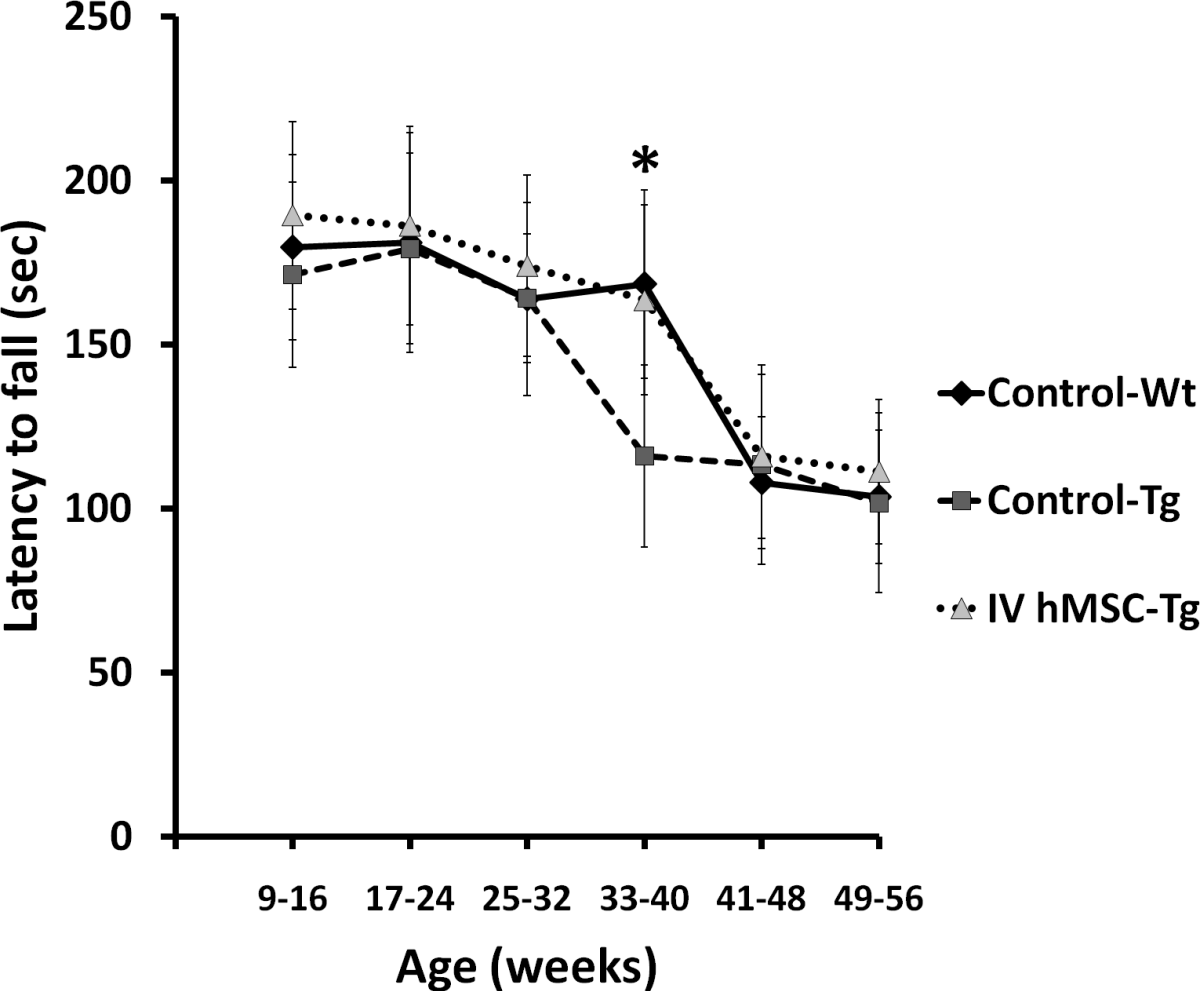
**Average of rotarod performance of mouse which received intravenous human mesenchymal stem cells transplantation at sequential periods**. Average of latency to fall from rotarod (in seconds) of mice after serial hMSCs implantation through intravenous injection was compared every 8 weeks. Rotarod performance of SCA2 transgenic mice (n = 14) was significantly improved at 33-40 weeks of age by serial intravenous hMSCs transplantation (*p = 0.012). The numbers of mice in Control-Wt and Control-Tg were 16 and 10, respectively. Statistical analysis between each group was performed by one-way ANOVA (p = 0.012).

Although the rotarod performance was not improved by intravenous MSC injection at 25-32 weeks of age, SCA2 transgenic mice which received intravenous MSC injection for four times had significantly better rotarod performance than Control-Tg mice at 33-40 weeks of age (Figure 4) (one-way ANOVA, p = 0.012). SCA2 transgenic mice which received intravenous hMSC injection also had similar rotarod performance with wild-type mice. This result suggested that intravenous transplantation of hMSCs via tail vein could ameliorate the deterioration of motor function in SCA2 transgenic mice.

### Rescue of Purkinje Cells by Transplanted hMSCs

**Figure 5:**
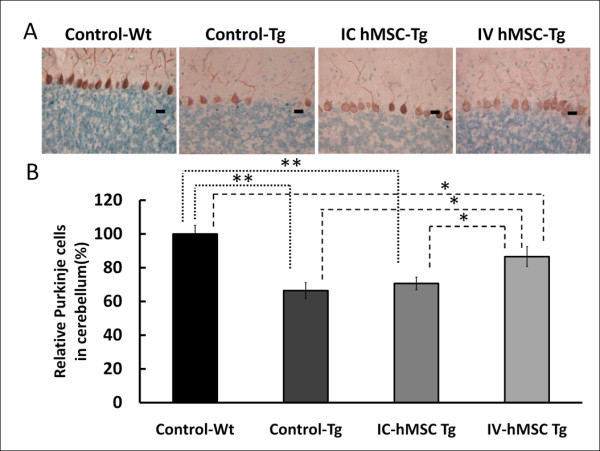
**Immunohistochemistry staining for murine Purkinje cells in cerebellum**. (A) Whole brain sections of wild-type mouse, SCA2 Tg mouse as control, and SCA2 transgenic mouse which received intravenous and intracranial human mesenchymal stem cells transplantation (4 μm) were processed by immunohistochemistry of calbindin D28K for Purkinje cells. Photographs were taken from the view of 100-folds microscopy and the scale bar was 40 μm. (B) Quantitative counting of calbindin D28K+ cells in cerebellum were compared to those of Control-Wt. Statistical analysis was performed by one-way ANOVA. (* p < 0.05; ** p < 0.001).

Loss of PCs had been noted by immunohistochemical stain of calbindin-28K, which was a protein specifically expressed in cytoplasm and dendritic processes of cerebellar PCs in SCA2 transgenic mice since age of 4 weeks [28]. Percentage of surviving PCs showed a progressive decline. At 24-27 weeks, PC number was reduced by 50-53% in SCA2 transgenic mice [28]. In our study, PC number (by visual impressions) in cerebellar sections of the IC-hMSC-Tg and IV-hMSC-Tg groups at 33-40 weeks of age was higher than in the Control-Tg group and similar with number in the Control-Wt group (Figure 5A). To obtain quantitative data supporting these visual impressions, the numbers of surviving PCs in the cerebellum of each group were estimated. Residual PCs in Control-Tg group accounted for 66.4 ± 4.7% of wild-type mice (100.0 ± 5.1%), while residual PCs in the IC-hMSC-Tg and IV-hMSC-Tg groups accounted for 70.7 ± 3.8% and 86.6 ± 5.9% (Figure 5B) (one-way ANOVA, p < 0.001). This result suggested that both serial intravenous and intracranial MSC transplantation had some neuroprotective effects on cerebellar PCs in SCA2 transgenic mice and intravenous MSC transplantation rescued more cerebellar PCs than intracranial transplantation (one-way ANOVA, p = 0.018).

### Grafted hMSCs in Murine Cerebellum and Cerebral Cortex

**Figure 6:**
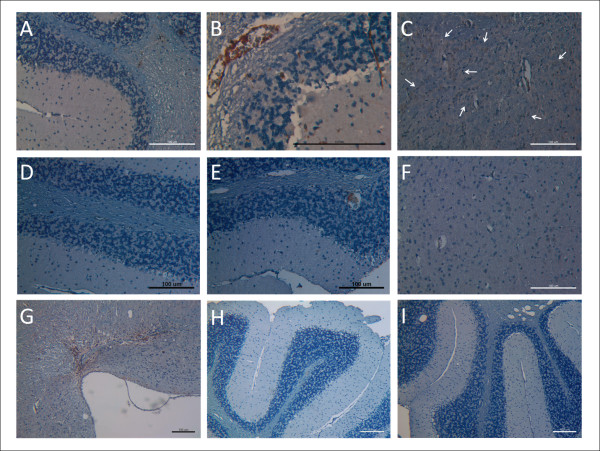
**Immunohistochemistry staining for human mesenchymal stem cells in whole brain sections of mice**. Whole brain sections of each mice (4 μm) were proceeded immunohistochemistry staining of β2 microglobulin for hMSCs. Photographs were taken from the view of 100, 200 or 400-folds microscopy and the scale bar was 100 μm. (A-C) In IV-hMSC-Tg group, hMSCs were located over the cerebellar white matter (A), molecular layer, and the lumens of blood vessels in white matter (B). Large clusters of grafted hMSCs were detected within cerebral cortex as arrows (C). (D-G) In IC-hMSC-Tg group, positive brown signals were not detected over cerebellar white matter, molecular layer, or the Purkinje cell layer (D), but limited to a few lumen of blood vessels (E) and a few scattered cells in cerebral cortex (F). Positive signals of hMSCs were detected over the injection site beneath the dorsal surface of medulla (G), which was adjacent to the cerebellum. (H, I) No signals were detected in the cerebellar sections of Control-Wt (H) and Control-Tg mice (I).

In IV-hMSC-Tg group, hMSCs which were positive for human β2 microglobulin signals were located in the cerebellar white matter (Figure 6A), molecular layer, and lumens of blood vessels in white matter (Figure 6B). Large clusters of grafted hMSCs were also detected in the cerebral cortex as arrows (Figure 6C). These data suggested that hMSCs which were transplanted via tail vein injection may extravasate intracranial vessels, and then migrate through white matter into cerebellar white matter, molecular layer, and cerebral cortex.

In IC-hMSC-Tg group, positive signals of hMSCs were not detected over cerebellar white matter, molecular layer, or Purkinje cell layer (Figure 6D), but limited to a few lumen of blood vessels (Figure 6E) and a few scattered cells in the cerebral cortex (Figure 6F). Positive brown IHC signals were also detected at the injection site beneath the dorsal surface of medulla, which was adjacent to the cerebellum (Figure 6G). No grafted cell adopted the morphological and immunohistochemical characteristics of PCs in either group. No IHC signals were detected in the cerebellar sections of Control-Wt (Figure 6H) and Control-Tg mice (Figure 6I), neither. Besides, no tumor formation was detected in the serial sections of cerebellums processed from six SCA2 transgenic mice which received intracranial and intravenous MSCs transplantation at time of sacrifice.

## Discussion

In this study, we investigate whether transplantation of hMSCs can rescue cerebellar PCs and ameliorate the deterioration of motor function in SCA in a preclinical animal model using SCA2 transgenic mice. After pre-test of intracranial trypan blue injection (Figure 1A) and SPIO-labeled hMSCs transplantation (Figure 2), SCA2 transgenic mice were serially transplanted with hMSCs for three times intracranially or four times intravenously (Figure 1B). Motor function of mice was evaluated by an acceleratng protocol of rotarod test every 8 weeks. Latency to fall on rotarod test of SCA2 transgenic mice which received serial intracranial hMSC transplantation of hMSCs failed to show significantly improved motor function (Figure 3). On the contrary, intravenous hMSCs transplantation significantly prolonged latency to fall at 33-40 weeks of age (Figure 4). IHC of serial cerebellar sections revealed that intravenous hMSC transplantation effectively rescued more cerebellar PCs than intracranial transplantation (Figure 5), which was compatible to rotarod performance of mice. In intravenous transplantation group, there were also more hMSCs which were positive for human β2 microglobulin signals in the cerebellum and cerebral cortex than in intracranial transplantation group (Figure 6).

At first, mouse was sacrificed to verify the intracranial presence of dye after trypan blue injection through foramen magnum into position of cerebellum (Figure 1A). Then SPIO-labeled hMSCs was transplanted intracranially and MR imaging of living mice was arranged to demonstrate the injection site (Figure 2). Low T2-intensity signals of injected SPIO-labeled hMSCs were found beneath dorsal surface of medulla, which was adjacent to cerebellum in MR imaging, and no evidence of major trauma or intracranial hemorrhage was observed. Therefore, intracranial and intravenous hMSCs transplantation proceeded as planned.

We found that rotarod performance of SCA2 transgenic mice was not significantly improved by serial intracranial hMSCs transplantation, and only a trend of better rotarod performance at 33-40 weeks of age (Figure 3). The limited number of transgenic mice which used in intracranial hMSC might probably result in bias in statistics. Moreover, injection site of intracranial transplantation was beneath dorsal surface of medulla, rather than the cerebellum, which made the distance of hMSCs migration longer.

Rotarod performance of SCA2 transgenic mice was effectively improved at 33-40 weeks of age by serial intravenous transplantation of hMSCs via tail vein (Figure 4). Because previous study had shown that the majority of intravenously administered MSCs (*>*80%) accumulated immediately in the lungs and were cleared with a half-life of 24 h [29], four times of intravenous transplantation which delivered larger cell dose of hMSCs were given in our study. There was no risk of causing tissue trauma or intracranial hemorrhage for intravenous transplantation, either. MSCs were also delivered intravenously in animal models of double toxin-induced multiple system atrophy-parkinsonism [30], lupus nephritis [31], and clinical trials of ischemic stroke [23], multiple system atrophy [20], and various diseases [32] with encouraging results.

IHC showed a marked decline of PC number (66.4% of wild-type mice) in Control-Tg mice (Figure 5A), which was previously demonstrated in a mouse model [28] and an autoposy report [33]. More cerebellar PCs were found in cerebellar sections of mice which received intracranial and intravenous hMSCs transplantation by visual impression (Figure 5A). After counting the numbers of surviving PCs, we found that intravenous hMSCs transplantation significantly rescued more cerebellar PCs (86.6% of wild-type mice) in SCA2 transgenic mice than intracranial transplantation (70.7% of wild-type mice, p = 0. 018) (Figure 5B). This result was compatible to rotarod performance of transplanted mice. However, the neuroprotective effects of hMSC transplantation might be offset by aging effect, since no difference of rotarod performance among all groups (including wild-type mice) was noted after 40-47 weeks of age. To elucidate the aging effect, the histological examinations and IHC at serial time points will be checked in the future experiments.

To further elucidate the engraftment of transplanted hMSCs in mice brain, IHC using specific antibodies against human beta2 microglobulin was performed on murine whole brain sections (Figure 6). There were more grafted hMSCs in the cerebellum (Figure 6A and 6B) and cerebral cortex (Figure 6C) in intravenous transplantation group than in intracranial transplantation group. Furthermore, cluster of grafted hMSCs in the cerebral cortex may also contribute to the better motor function of mice in intravenous transplantation group, since degeneration may be encountered in the cerebral cortex in SCA2 patients [5,6,8]. Local tissue damages to medulla may be caused by invasive procedures of serial intracranial transplantation (Figure 6G). Stereotaxic implantation should be considered to improve target localization and minimize complications in the future experiments. All these findings suggested that intravenous hMSCs transplantation was more effective to ameliorate motor function deterioration of transgenic SCA2 mice than intracranial transplantation.

Systemically administered MSCs home to sites of ischemia or injury and may either transdifferentiate into exogenous functional neurons or provide neurotrophic factors for endogenous cells [19,34]. No grafted cell adopted the morphological and immunohistochemical characteristics of cerebellar PCs in this mouse model. As a result, neuroprotective effects of intravenous hMSCs transplantation in this study mainly resulted from neurotrophic factors or direct cell contact with host cells, not transdifferentiation. Two transgenic mouse model of SCA1 [10] and cerebellar ataxia [11] reported the similar findings. Many recent clinical studies which adopt systemically administered MSCs also implicate paracrine signaling as the primary mechanism of action [32].

Although clinical trials of MSC transplantation have shown no major adverse events over the past 10 years of testing, recent preclinical studies have stressed potential long-term risks associated with MSC therapy that may not be observable in the short follow-up time period. These long-term risks include potential maldifferentiation, immunosuppression, and instigation of malignant tumor growth by directly promoting tumor growth, metastasis, and angiogenesis [32]. For example, when administered in immunocompromised mice by systemic injection, MSCs created microemboli and subsequently form osteosarcoma-like pulmonary lesions [35]. No tumor formation was detected in the serial sections of cerebellums and medulla processed from six SCA2 transgenic mice which hMSCs had been transplanted at time of sacrifice in our study (Figure 6). More preclinical and clinical studies are still needed to evaluate the safety issues of MSC transplantation.

## Conclusions

In summary, present study demonstrated that intravenous transplantation of hMSCs effectively improved rotarod performance of SCA2 transgenic mice and delayed the onset of motor function loss by better engraftment of hMSCs in brain tissues and rescuing cerebellar PCs from cell death, possibly through release of neurotrophic factors or direct cell contact with host cells; while intracranial transplantation only rescued a smaller portion of PCs and failed to improve motor function. Together, transplantation of hMSCs can indeed delay the onset as well as to improve the motor function of SCA2 transgenic mice. Results of this preclinical study strongly support further exploration of the feasibility to transplant hMSCs for SCA patients.

## Competing interests

The authors declare that they have no competing interests.

## Authors' contributions

YKC carried out the hMSCs culture, cell transplantation and rotarod test, performed the statistical analysis and drafted the manuscript. JHH and BWS provided the transgenic mice and participated in the design of the study. MHC took care of the animals and carried out the hMSCs culture, cell transplantation, MRI study and rotarod test. YHC and YFC carried out immunohistochemical stain of cerebellar sections and counting of Purkinje cells. WHM and CYT carried out immunohistochemical stain of whole brain sections and identification of engrafted human cells. OKL conceived of the study and participated in its design and coordination. All authors read and approved the final manuscript.
